# Escolha de Dupla Antiagregação na Doença Arterial Coronariana: Apenas uma Questão de Equilíbrio entre a Carga Isquêmica e o Risco de Sangramento?

**DOI:** 10.36660/abc.20240695

**Published:** 2024-12-20

**Authors:** Maria Cristina Almeida, Marildes Luiza de Castro, Larissa Espíndola

**Affiliations:** 1 Centro Universitário de Belo Horizonte Belo Horizonte MG Brasil Centro Universitário de Belo Horizonte, Belo Horizonte, MG – Brasil; 2 Universidade Federal de Minas Gerais Faculdade de Medicina Belo Horizonte MG Brasil Universidade Federal de Minas Gerais - Faculdade de Medicina, Belo Horizonte, MG – Brasil; 3 Hospital Santa Izabel Salvador BA Brasil Hospital Santa Izabel, Salvador, BA – Brasil

**Keywords:** Dupla Antiagregação Plaquetária, Stent, Isquemia Miocárdica

A síndrome coronariana aguda ou crônica é uma das ameaças mais importantes à saúde pública. Devido às alterações estruturais e/ou funcionais das artérias coronárias e/ou da microcirculação, a doença pode culminar em desequilíbrio entre a demanda miocárdica e o suprimento de sangue, resultando em isquemia.

A prevenção primária de eventos isquêmicos coronários agudos baseada em diminuir o risco de oclusão da artéria coronária é uma medida muito importante. A prevenção secundária após a revascularização miocárdica por intervenção coronária percutânea (ICP) com *stent* é importante para reduzir o risco de trombose no *stent*, eventos isquêmicos e infarto do miocárdio (IM), particularmente logo após o implante de *stent*. Muitos protocolos foram estudados, mas a a dupla antigregação plaquetária (DAPT) com aspirina e um inibidor P2Y12 potente é o padrão atual de tratamento após a ICP.^1^

**Figura 1:**
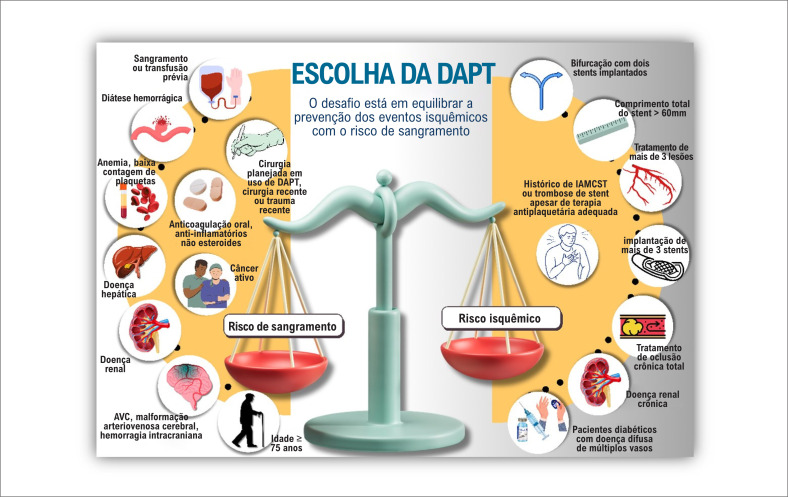
Escolha da dupla antigregação plaquetária (DAPT): o desafio está em equilibrar a prevenção dos eventos isquêmicos com o risco de sangramento. IAMCST: Infarto agudo do miocárdio com elevação do segmento ST; AVC: acidente vascular cerebral.

A intensidade e a duração ideal da DAPT após ICP continuam controversas, especialmente pela associação com um aumento no risco de sangramento. Por outro lado, a DAPT está associada a uma menor ocorrência de IM após a ICP em comparação à monoterapia com aspirina ou com inibidor P2Y12.^2^ Assim, a decisão sobre qual estratégia escolher na DAPT baseia-se na avaliação da carga isquêmica e do risco de sangramento (Figura 1).

O risco de sangramento é proporcional à intensidade e à duração da DAPT, mas o perfil individual de risco e benefício ajuda no rastreio de indivíduos em alto risco de sangramento, de acordo com o escores de risco como ARC-HBR e PRECISE-DAPT. A avaliação da carga isquêmica de acordo com escores de risco de isquemia como SYNTAX 2, GRACE, TIMI e DAPT, na presença de situações como idade avançada, diabetes mellitus e dislipidemia deve ser considerada.^3^

Os escores dão direcionamento aos médicos em ajustar a duração e a intensidade da DAPT aos perfis individuais dos pacientes, melhorando os desfechos clínicos. O risco isquêmico é mais alto nos primeiros meses após a ICP e diminui posteriormente, ao passo que o risco de sangramento tende a se manter consistentemente elevado ao longo do tempo.^4^

O escore PRECISE-DAPT é comumente utilizado; um escore mais alto indica um risco de sangramento elevado, sugerindo que a DAPT por períodos mais curtos ou o uso de agentes menos potentes pode ser mais seguro. O ARC-HBR é outra ferramenta amplamente aceita para identificar pacientes em alto risco de sangramento. Esses critérios são validados pela capacidade de predizer um risco anual de sangramento maior de 4% ou mais, segundo classificação BARC 3 ou 5.^5^

**Figura 2:**
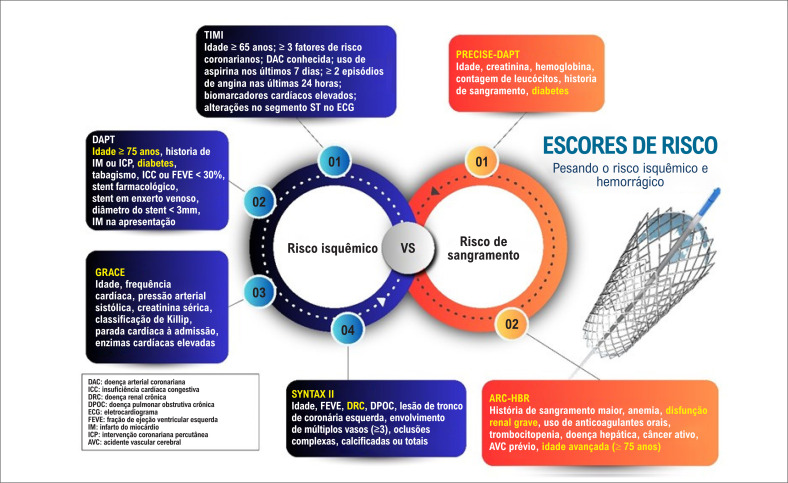
Escores de risco: risco de isquemia e de sangramento.

O risco de isquemia pode ser avaliado usando os escores DAPT e SYNTAX II, que ajuda a determinar se um paciente se beneficiaria de terapia prolongada ou mais intensiva.^6^ Quando um alto risco isquêmico é identificado, os benefícios da DAPT estendida devem ser pesados contra os riscos de sangramento indicados pelo ARC-HBR.^6^ O escore GRACE pode fornecer um *insight* para o risco isquêmico pós-síndrome coronariana aguda, ajudando a direcionar a necessidade de estratégias antiplaquetárias mais agressivas (Figura 2).

A interação entre o risco de isquemia e o risco de sangramento é complexo, e a tomada de decisão clínica deve ser individualizada.^4^

A modulação da terapia antitrombótica é crucial para se atingir o ponto entre segurança e eficácia do tratamento. As diretrizes não são claras sobre a melhor estratégia para pacientes com alto risco de sangramento, e o uso de escores pode ser útil.

Em pacientes com alto risco isquêmico e baixo risco de sangramento, estender a DAPT além de 12 meses pode ser benéfico. Para pacientes com um alto risco de sangramento e um baixo risco isquêmico, uma mudança na terapia diminuindo-se a duração da DAPT ou alterando-se para agentes menos potentes pode ser apropriado.^1,6,7^

Os escores de risco são ferramentas valiosas, porém, o julgamento clínico, preferências do paciente, e características individuais devem ajudar na decisão final sobre a duração e a intensidade da DAPT.^7^
