# Supplementary material for: Escolha de Dupla Antiagregação na Doença Arterial Coronariana: Apenas uma Questão de Equilíbrio entre a Carga Isquêmica e o Risco de Sangramento?
Source: Arq Bras Cardiol. 2024 Dec 20;121(11):e20240695. [Article in Portuguese] doi: 10.36660/abc.20240695 (PMC11809845; doi:10.36660/abc.20240695)
Supplement: Translation

# Translation: The Choice of Antiplatelet Dual Combination in Coronary Artery Disease: Is this Just a Question of Balance between ischemic Burden and Bleeding Risk?

**DOI:** 10.36660/abc.20240695i

**Authors:** Maria Cristina Almeida, Marildes Luiza de Castro, Larissa Espíndola

**Affiliations:** 1 Belo Horizonte MG Brazil Centro Universitário de Belo Horizonte, Belo Horizonte, MG – Brazil; 2 Belo Horizonte MG Brazil Universidade Federal de Minas Gerais - Faculdade de Medicina, Belo Horizonte, MG – Brazil; 3 Salvador BA Brazil Hospital Santa Izabel, Salvador, BA – Brazil

**Keywords:** Dual Anti-Platelet Therapy, Stent, Myocardial Ischemia

Acute or chronic coronary syndrome is one of the most important threats to public health. Because of structural and/or functional alterations of the coronary arteries and/or microcirculation, it can culminate in disbalance between myocardial demand and blood supply resulting in ischemia.

Primary prevention of acute coronary ischemic events based on lowering the risk of coronary artery occlusion is a very important measurement. Secondary prevention after myocardial revascularization by percutaneous coronary intervention (PCI) with stent is important to reduce the risk of stent thrombosis, ischemic events and myocardial infarction (MI), particularly early after stenting. Many protocols have been studied, but the dual antiplatelet therapy (DAPT) with aspirin plus a potent P2Y12 inhibitor is the current standard of care after PCI.^1^

The intensity and optimal duration of treatment with DAPT after PCI remains controversial, especially because it is associated with an increased risk of bleeding. On the other hand, DAPT is associated with less MI after PCI in comparison with aspirin or P2Y12 inhibitor monotherapy.^2^ Therefore the decision on which strategy to choose in DAPT treatment is based on the evaluation of ischaemic burden and bleeding risk (Figure 1).

**Figure 1:** Dual antiplatelet therapy (DAPT) choice: the challenge lies in balancing the prevention of ischemic events with the risk of bleeding; STEMI: ST-elevation myocardial infarction.

The bleeding risk is proportional to the intensity and duration of DAPT, but individual risk-benefit profile helps to screen who are at high bleeding risk (HBR), according to risk scores as ARC-HBR and PRECISE-DAPT. The evaluation of ischemic burden according to ischemic risk scores as SYNTAX 2, GRACE, TIMI and DAPT in presence of situations as advanced age, diabetes mellitus and dyslipidemia should be considered.^3^

These scores guide clinicians in tailoring DAPT duration and intensity to individual patient profiles, improving clinical outcomes. The ischemic risk is highest in the first few months after PCI and decreases thereafter, while the bleeding risk tends to remain consistently elevated over time.^4^

PRECISE-DAPT score is commonly used; a higher score indicates a HBR, suggesting that shorter durations of DAPT or less potent agents may be safer. ARC-HBR is another widely accepted tool designed to identify patients with HBR. These criteria are validated by the ability to predict a 4% or greater annual risk of major bleeding, as per the BARC 3 or 5 bleeding classifications.^5^

Ischemic risk can be assessed using DAPT score and SYNTAX II score which helps determine if a patient would benefit from prolonged or more intensive therapy.^6^ When high ischemic risk is identified, the benefits of extended DAPT must be weighed against the bleeding risks indicated by the ARC-HBR.^6^ GRACE score can provide insight into post-acute coronary syndrome ischemic risk, helping to guide the need for more aggressive antiplatelet strategies (Figure 2).

**Figure 2:** Risk scores: ischemic risk and bleeding risk.

The interplay between ischemic and bleeding risk is complex, and clinical decision-making must be individualized.^4^

Modulation of antithrombotic therapy is crucial to achieve the point between safety and efficacy of the treatment. The guidelines are not clear about the best strategy for HBR patients and utilizing scores may be useful.

In patients at high ischemic risk and low bleeding risk, extending DAPT beyond 12 months may provide substantial benefit, and patients with a HBR and low ischemic risk, a change in therapy by shortening DAPT duration or switching to less potent agents may be appropriate.^1,6,7^

Risk scores are valuable tools, but clinical judgment, patient preferences, and individual characteristics should guide the final decision on DAPT duration and intensity.^7^
